# The Effect of Radon Concentration on MS Prevalence: A Door-to-Door Survey in the Fault Zone in Afyonkarahisar, Turkey

**DOI:** 10.3390/toxics13090797

**Published:** 2025-09-19

**Authors:** Ülkü Türk Börü, Ahmet Yıldız, Metin Bağcı, Ayla Sandıkçıoğlu Gümüş, Elif Simin Issı, Furkan İncebacak, Hakan Acar, Cem Bölük

**Affiliations:** 1 Department of Neurology, University of Afyonkarahisar Health Sciences, Afyonkarahisar 03000, Türkiye; uturkboru@hotmail.com (Ü.T.B.); elifsiminissi@gmail.com (E.S.I.); hakanacar4516@hotmail.com (H.A.); 2 Department of Geological Engineering, Faculty of Engineering, University of Afyon Kocatepe, Afyonkarahisar 03200, Türkiye; ayildiz@aku.edu.tr (A.Y.); metbagci@gmail.com (M.B.); 3 Department of Physics, Faculty of Science and Literature, University of Afyon Kocatepe, Afyonkarahisar 03200, Türkiye; sandikci@aku.edu.tr; 4 Department of Neurology and Clinical Neurophysiology, Palmiye Hospital, Hatay 31200, Türkiye; cem_boluk@hotmail.com

**Keywords:** radon gas, multiple sclerosis prevalence, faulty zone, environmental factors

## Abstract

**Background:** Despite the identification of various environmental factors that increase the risk of multiple sclerosis (MS), the effects of many factors on the etiology of MS remain to be elucidated. In this study, we aimed to investigate the effects of radon, a factor previously studied in relation to various other neurodegenerative diseases, on the epidemiology of MS. **Methods:** A door-to-door field study was conducted in residential areas with relatively high and low radon gas concentrations to determine the prevalence of MS. The study area comprises the Bolvadin and İhsaniye regions, which have different geological characteristics, such as seismic activity, active faults, and distributions of volcanic rocks. CR-39 detectors, with an accepted limit of 300 Bq/m^3^, were utilized to measure radon gas concentrations. During the screening field, the patients diagnosed with multiple sclerosis were confirmed with their hospital records. Mc Donald’s revised diagnostic criteria were used for multiple sclerosis diagnosis. **Results:** The regions were grouped into higher radon areas and lower radon areas. The İhsaniye city center, Kayıhan, Kemerkaya, Döğer, and Bolvadin city center were classified as higher radon regions, whereas Dişli, Yaylabağı, Gazlıgöl, and Özburun were identified as lower radon regions. A total of 40,841 individuals were surveyed in the field. The crude MS prevalence was 41.8/100,000 in settlements with high radon gas concentrations and 20.5/100,000 in settlements with low radon gas concentrations. **Conclusions:** In this study, we revealed that the prevalence of MS was greater in settlements with high radon gas concentrations than in settlements with low radon gas concentrations. These results demonstrated that radon gas is an important environmental risk factor in the etiopathogenesis of MS.

## 1. Introduction

Multiple sclerosis (MS) is an immune-mediated disease in which the brain and spinal cord can be affected. T and B cells together contribute to secondary axonal damage in addition to myelin loss. The etiology of MS still needs to be elucidated. The pathogenesis is multifactorial and is associated with both genetic and environmental factors [1]. Environmental factors, such as geography, latitude, sunlight, vitamin D, smoking, and infections, have been identified as contributing elements. Additionally, studies have been conducted on air and environmental pollution as potential factors [2,3]. There are also some data concerning geochemical triggers of MS: It was recorded that the prevalence rate of MS is higher in areas with lead-enriched bedrock and soils [4,5], whereas another study established links between the morbidity rate of this disease and indoor radon [6].

Neurodegenerative diseases, such as MS, amyotrophic lateral sclerosis, Alzheimer’s disease, and Parkinson’s disease, have been hypothesized to potentially result from radon gas exposure. When an individual inhales air containing radon, the majority of the inhaled radon is exhaled immediately. However, a small portion remains trapped in the lungs and subsequently enters the circulatory system. Through this system, radon is transported to various tissues with high lipid contents, including the brain, bone marrow, and nervous system. Over time, these tissues may sustain damage due to the toxic and radioactive effects of radon exposure [7,8,9].

Radon and its decay products have previously been investigated as potential risk factors for neurodegenerative diseases. Gómez-Ancá and Boris-Dios conducted a systematic review that identified 10 studies examining the association between radon exposure and neurodegenerative diseases. Among these 10 studies, 5 specifically explored the relationship between radon gas and the prevalence or incidence of MS. Three of these studies reported a positive association, whereas the remaining two found no significant link. Additionally, a few studies have indicated a possible relationship between radon concentration and conditions such as motor neuron diseases and Alzheimer’s disease [9,10].

A significant positive correlation has been observed between MS rates and indoor radon (Rn) levels [9]. In Norway, radon exposure levels have been found to be positively correlated with motor neuron disease mortality and MS rates [11,12].

Radon (^222^Rn) is a naturally occurring radioactive inert gas that forms as a decay product of radium in uranium-containing rocks and soils. It contributes to approximately half of the radiation dose absorbed by the general population. Radon readily migrates from rocks and soil into the atmosphere through processes such as diffusion and convection [13]. This gas is present at lower concentrations in outdoor air than in indoor environments. In particular, poorly ventilated rooms on the lower floors of buildings tend to have relatively high concentrations of radon [14].

The most significant radon isotope, ^222^Rn, decays via α-emission, with a half-life of 3.8 days, progressing through isotopes such as ^218^Po and ^214^Bi (both α-emitters) to ^210^Po, eventually forming the stable lead isotope ^206^Pb. These heavy metal decay products are highly toxic and readily adsorb onto atmospheric particles and lung tissue. Inhalation of these products, along with ^222^Rn and its progeny, accounts for the majority of the radiation dose absorbed by the respiratory system, posing a significant health hazard [15].

### 1.1. Relationship Between Radon, Tectonic Structure, and Lithology

Tectonics and lithology are the most important geological features that affect the concentration of radon, its distribution in the Earth’s crust, and its transport [16].

Faults, fractures, and fissures in bedrock serve as primary pathways for the escape of radon gas [17,18,19].

Regions where faults, fractures, and fissures are present are known to present high concentrations of ^222^Rn in surface soil and groundwater, including springs associated with faults [20,21,22,23,24].

In numerous instances, elevated radon levels have been utilized as indicators of faults and large fractures within bedrock [25].

Some studies suggest that the presence of faults does not necessarily result in elevated radon concentrations in air and water. Faults must have experienced seismic activity within the last hundred years to increase radon concentrations, and recent seismic activity is necessary [26].

Earthquakes can activate existing faults and lead to the formation of new faults. They also increase the porosity and permeability of the surrounding rock and soil, facilitating the migration of gases such as radon [19]. This process results in increased radon release from bedrock and soil [25,26,27].

Since radon is formed by the decay of radioactive elements such as ^238^U and ^232^Th and their derivatives, radon has a high release rate in environments where rocks (granite, tuff, schist, phosphatic rocks, etc.) containing high concentrations of uranium and thorium. However, basalt, limestone, and sandstone have low uranium relase rate [16].

### 1.2. Tectonic Structure of Afyonkarahisar

**Figure 1:**
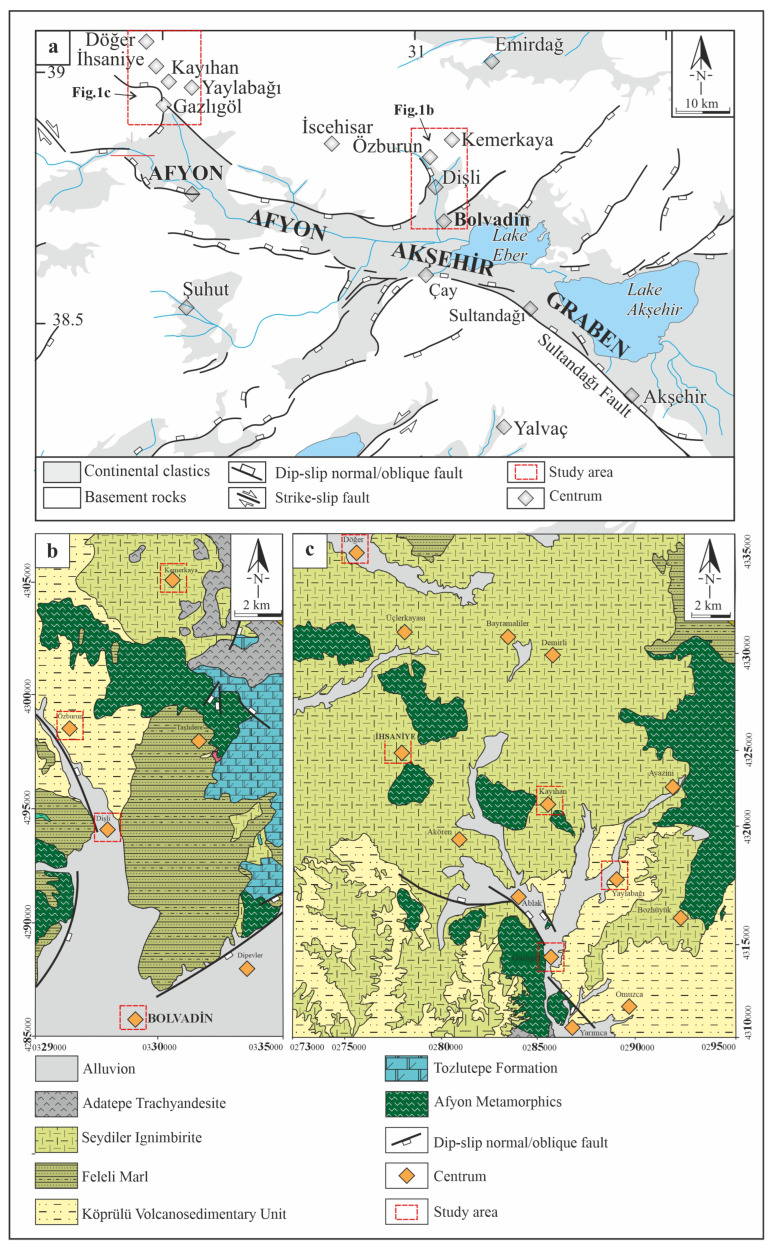
Geological map of the study area: (**a**) Afyon-Akşehir graben system [30]; (**b**) Bolvadin region [31]; (**c**) İhsaniye region [32,33].

Afyonkarahisar is located at the intersection of the Aegean and Central Anatolian regions, an area influenced by the extensional tectonism of Western Anatolia. This tectonic activity has resulted in earthquakes of varying magnitudes over time. Notable examples include the 1995 Dinar earthquake (magnitude 6.1), the 2000 and 2002 Sultandağı earthquakes (magnitudes 6.0 and 6.5), and the 2002 Çay earthquake (magnitude 6.0), all of which caused significant destruction. Furthermore, the region’s active tectonic structure and geological characteristics have led to the formation of geothermal resources in various parts of Afyonkarahisar. Considering the movement of faults and the effects of different rock ty pes on radon gas concentrations, we investigated the relationship between radon gas levels and MS in residential areas within the Bolvadin and İhsaniye regions of Afyonkarahisar, which exhibit distinct geological and seismic properties (Figure 1a) [28,29,30,31,32,33]. 

Our hypothesis is that radon gas may be an etiological factor in the pathogenesis of MS. Elevated radon gas concentrations in seismically active and volcanic areas may contribute to an increased prevalence of MS. To test this hypothesis, we investigated the relationship between radon gas concentrations and MS prevalence in the Bolvadin and İhsaniye regions (Afyonkarahisar).

## 2. Materials and Methods

### 2.1. Determination of the Study Area

The Bolvadin and İhsaniye regions in Afyonkarahisar, characterized by distinct geological characteristics, such as seismic activity, active faults, and distributions of volcanic rocks, were selected as the study area. The Bolvadin region includes the city center of Bolvadin and the towns of Dişli, Özburun, and Kemerkaya, whereas the İhsaniye region comprises the city center of İhsaniye and the towns of Gazlıgöl, Kayıhan, Yaylabağı, and Döğer (Figure 1a). The city center of Bolvadin is classified as a tectonically active region because of its location in a seismically active area, where coseismic ruptures and aseismic creeps associated with the Bolvadin Fault have been observed in recent years. In contrast, the İhsaniye region is considered a tectonically inactive area due to its weak seismic activity and its distance from major active faults, such as the Erkmen Fault and the Çobanlar Fault Zone [30,31,32,33]. The demographic, geographic, and economic conditions of the two regions where this study was conducted are very similar. These regions are rural areas with characteristics of an agriculturally based rural area between Central Anatolia and the Aegean region. There are no immigrant populations in the region; it consists of a homogeneous Turkish population. 

### 2.2. Determination of Sample Size

The total population consists of 74,127 individuals, with 63,863 residing in five settlements characterized by high seismic activity and 10,264 residing in four settlements with low seismic activity. To calculate the minimum sample size for the five settlements with high tectonic activity, the formula n = P.Q. Z^2^_α_/d^2^, as proposed by National Education Assosiaton (NEA) and Sekaran, was utilized [34,35].

In the formula, the parameters were set as P = 50% and Q = 50%. The significance level was set at α = 0.01, corresponding to a theoretical value of Z_0.01_ = 2.58, with a sampling error of d = 0.75%. On the basis of these parameters, a minimum of 29,584 individuals should be surveyed in settlements with high seismic activity. Considering potential data inaccuracies, a total of 31,417 individuals (63,863/31,417 = 2.03) were targeted for screening. Systematic sampling was employed, where every second house was visited, and surveys were conducted. Additionally, the aim was to survey the entire population of 10,264 individuals in the four settlements with low radon gas concentrations.

### 2.3. Geology of the Study Area

The Bolvadin region is situated within the Afyon-Akşehir graben, which formed tectonically in association with the Akşehir-Simav fault system. In the Bolvadin city center, a seismic fault has developed due to the influence of the active Bolvadin Fault located along the northern boundary of the Afyon-Akşehir graben. The Paleozoic Afyon metamorphic rocks in the Afyon Zone are the basement rocks in both the Bolvadin and İhsaniye regions [30].

Alluvium is widespread in the Bolvadin city center, while Dişli features a combination of alluvium, Feleli marl, and the Köprülü volcano-sedimentary unit. Özburun is dominated by the Feleli marl, and Kemerkaya is characterized by the Adatepe trachyandesite. The Bolvadin city center is built primarily on alluvium. Additionally, alluvium, the Feleli marl, Köprülü volcano-sedimentary unit, Seydiler ignimbrite, and Adatepe trachyandesite are widely distributed in the Dişli, Özburun, and Kemerkaya regions (Figure 1b).

The İhsaniye region lies north of the Akşehir-Simav fault system and is located in an area of low seismic activity (Figure 1). The Gazlıgöl and Yarımca faults are the most prominent tectonic structures in the İhsaniye region (Figure 1a). Although it was shown as an active fault on the map [36,37], these faults were evaluated as inactive faults in our study because of the absence of seismic activity on the Gazlıgöl and Yarımca faults in recent years. The İhsaniye city center and the towns of Kayıhan and Döğer are situated on ignimbritic rocks. Moreover, Afyon metamorphics, the Köprülü volcano-sedimentary unit, and alluvium are observed in the Gazlıgöl and Yaylabağı areas (Figure 1c) [30,31,32,33,38]. 

### 2.4. Radon Measurement and Data Analysis

#### 2.4.1. CR-39 Detector

**Figure 2:**
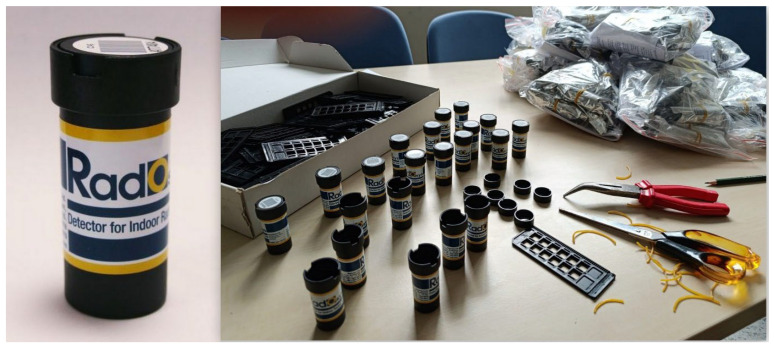
The plastic covers of the CR-39 detectors were opened, and the detectors were collected.

In this study, radon activity concentrations were measured using a passive radon measurement method with the Radosys system and CR-39 detectors obtained from the Turkish Energy, Nuclear, and Mineral Research Agency (TENMAK, Ankara, Türkiye). The system comprises CR-39 solid-state nuclear track detectors (SSNTDs), a bath unit for the chemical treatment of the detectors, and a computer-aided optical reader for trace counting. The most widely used method for determining indoor radon concentrations is alpha particle counting with nuclear track detectors. Figure 2 shows the CR-39 detectors and the removal of their covers after collection from residential homes.

#### 2.4.2. Distribution and Collection of CR-39 Detectors

To assess the spatial variation in indoor radon concentrations, the detectors were distributed across the study area, ensuring that one detector was placed every 7 hectares. At the measurement locations, the detectors, wrapped in aluminum foil, were carefully removed from their packaging and positioned within the living spaces of dwellings. Care was taken to ensure proper placement: the detectors were kept away from electronic devices, stoves, and radiators, not in direct contact with walls, shielded from direct sunlight, placed at a height of 2 m from the ground, and remained undisturbed throughout the measurement period. The detectors recorded radon exposure in the environment for 60 days, after which they were collected and rewrapped in aluminum foil to prevent further air exposure.

#### 2.4.3. Analysis of Detectors

The collected detectors were transported to the TENMAK Laboratory for evaluation. Indoor radon activity concentrations were calculated in Bq/m^3^ using the following formula:

$$C_{Rn}=CF\times \left(\frac{\rho_{Tracks}}{46.8}\right)\times \left(\frac{1000}{T}\right)$$

where CRn represents the indoor radon activity concentration (Bq/m^3^), CF represents the calibration factor, ρTracks represents the track density (number of tracks per cm^2^), and T represents the detector exposure time (in hours). This formula incorporates standard parameters to ensure accurate calculations of radon concentrations in indoor environments [39,40].

A random selection of detectors in the laboratory for calibration and sensitivity testing is subjected to radon exposure using a standard radium source, serial number NIST SRM 4973. The irradiation value is read from the traceable Alphaguard device. The results obtained by completing the chemical etching and track counting processes on the exposed detectors are calculated using the equation below and used as the calibration factor for that series.

E = D × CF

E: Irradiation Value;
D: Trace Intensity (value found from the reader in the laboratory);
CF: Calibration Factor.

### 2.5. Informed Consent

Written consent was obtained from all the participants during the survey. Adequate time was given to participants to evaluate their participation.

### 2.6. Patient Screening in the Field

To conduct the survey, three teams were formed, each consisting of one neurology resident and four surveyors. The teams began administering surveys in one settlement and moved on to the next after completing the work in the first settlement. The study was conducted between 1 November 2022 and 1 January 2023. During this period, radon detectors were placed in dwellings and left for two months. At the end of the second month, the detectors were collected and sent to the TENMAK Laboratory for analysis.

The fieldwork was carried out between 10:00 AM and 6:00 PM. In Bolvadin, the largest settlement, the goal was to reach half of the total population. To achieve this goal, every second household was visited, and all members of the household were asked to complete the survey. In smaller towns and villages outside Bolvadin, all households were visited. For young children, individuals who were not able to communicate, and elderly residents, surveys were completed by asking adult family members.

The survey form used in this study included all MS symptoms identified in previous prevalence studies and utilized a Turkish-validated screening form [41].

In the survey, all individuals who answered “yes” to any MS symptom question were examined onsite by the neurology resident. Those suspected of having MS were invited to the Neurology Clinic of Afyonkarahisar Health Sciences University Hospital for further evaluation. At the clinic, these individuals underwent a detailed re-examination, and their information was compared with hospital records to confirm the diagnosis. Demographic data, initial symptoms, neurological examination findings, and radiological and laboratory results of the patients were recorded. Mc Donald’s revised diagnostic criteria were used for multiple sclerosis diagnosis [42]. The STROBE guideline was followed in reporting this study [43].

### 2.7. Statistical Analysis

Data were recorded electronically, and statistical analyses were performed using SPSS 21.0 (version 21.0, SPSS Inc., Chicago, IL, USA) for the calculation of frequency distributions and percentages.

### 2.8. Ethical Approval

All the methods were performed in accordance with the relevant local regulations and guidelines of the ethical commission. Ethical approval was obtained from the local ethics committee of Afyonkarahisar Health Sciences. Ethics committee approval date and number: 10/2023, 414. In addition, permission was granted by local authorities.

## 3. Results

### 3.1. Distribution of Radon Concentrations

**Figure 3:**
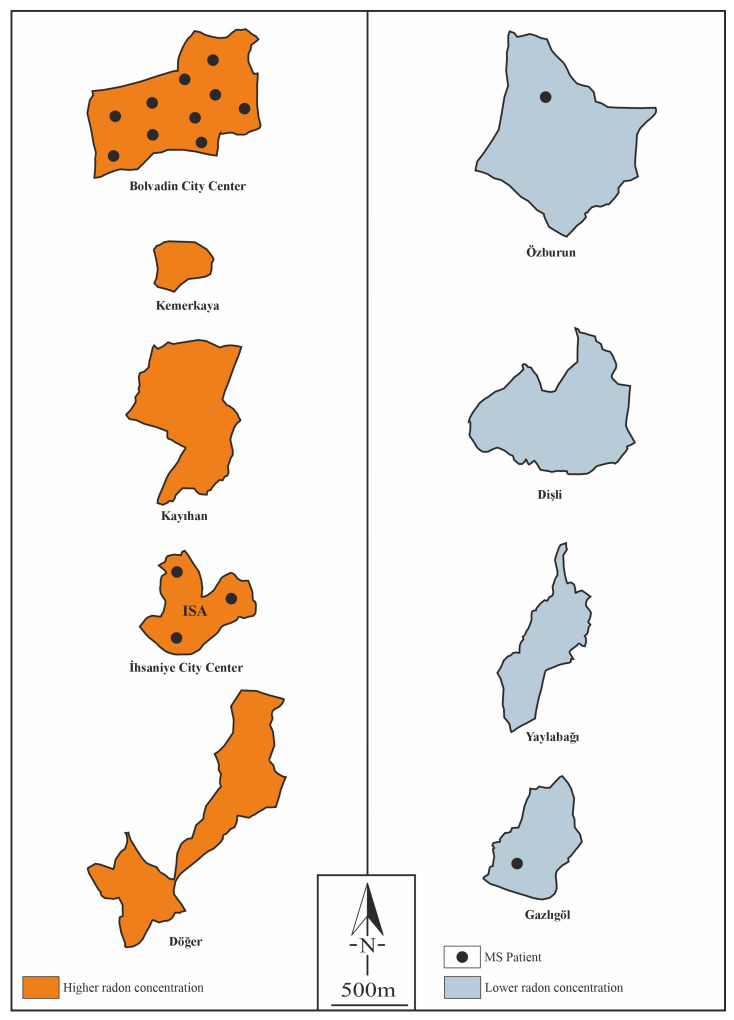
Radon and MS distributions in the regions of the study area [40].

In the study area, the minimum radon concentration was 12 Bq/m^3^ in the Gazlıgöl region. The radon concentrations in other locations were as follows: İhsaniye city center, 1878 Bq/m^3^; Kayıhan, 1688 Bq/m^3^; Kemerkaya, 1603 Bq/m^3^; and Bolvadin city center, 1325 Bq/m^3^ (Table 1). On the basis of the ICRP reference radon value of 300 Bq/m^3^ [44], the regions were grouped into higher and lower radon areas, as shown in Figure 3. The İhsaniye city center, Kayıhan, Kemerkaya, Döğer, and Bolvadin city center were classified as higher radon regions, whereas Dişli, Yaylabağı, Gazlıgöl, and Özburun were identified as lower radon regions. 

### 3.2. Prevalence of MS

The population of the five settlements with high radon gas concentrations was 63,863. According to the sample size, 31,417 individuals were targeted for screening. Among them, 327 persons declined to participate in the survey, resulting in a total of 31,090 participants being screened.

Among the four settlements with low seismic activity, the total population was 10,264. A total of 9751 individuals completed the survey, whereas 513 individuals refused to participate. In total, the refusal rate was 2%, indicating a high overall participation rate.

### 3.3. Patient Characteristics

In the five regions with radon gas concentrations exceeding 300 Bq/m^3^, a total of 13 MS patients were identified. In contrast, in the four regions with lower radon gas concentrations (below 300 Bq/m^3^), 2 MS patients were identified. The prevalence of MS in the high-radon regions was 41.8 per 100,000, whereas in the low-radon regions, the prevalence was 20.5 per 100,000.

The prevalence of MS in the high-radon regions was found to be nearly twice as high as that in the low-radon regions. A total of 15 MS patients were identified. The age range of the patients was 24 to 69 years, with a mean age of 42.5 ± 15.7 years. The female-to-male ratio among the patients was 11:4. The average age of disease onset was 30.7 ± 8.3 years, and the mean disease duration was 10.0 ± 10.9 years. The average EDSS (Expanded Disability Status Scale) score was 3.4 ± 1.8. Among the 15 patients, the distribution of MS subtypes was as follows: 11 patients had relapsing–remitting MS (RRMS), 3 patients had secondary progressive MS (SPMS), and 1 patient had primary progressive MS (PPMS). The clinical and demographic findings of the patients are shown in Table 2. The initial symptoms of the disease were categorized as follows: pyramidal symptoms in 4 patients, brainstem symptoms in 2 patients, cerebellar symptoms in 3 patients, sensory symptoms in 5 patients, cognitive dysfunction in 4 patients, visual symptoms in 2 patients, and autonomic symptoms in 1 patient (Table 3).

## 4. Discussion

The present study revealed that the prevalence of MS was 41.8/100,000 in five settlements with radon gas concentrations above 300 Bq/m^3^ and 20.5/100,000 in areas with low seismic activity below 300 Bq/m^3^. This study further demonstrated that indoor radon gas concentrations are elevated in areas with high seismic activity, suggesting a potential underlying cause for the high prevalence of MS. These findings indicate that radon gas may be a contributing factor in the etiopathogenesis of MS, alongside other environmental and genetic elements.

In the literature, studies investigating the relationship between radon and MS are scarce. There are no studies investigating the relationship between radon gas and MS in Turkey. A recent study examined the risk of neurodegenerative diseases, including MS, associated with radon gas exposure in male miners. However, no dose–response relationships between cumulative radon exposure and Alzheimer’s disease or Parkinson’s disease have been reported [10].

In a study involving 25 MS patients and 21 control patients, radon detectors were placed for six months at different living levels within their homes. The time-weighted average levels of radon exposure were calculated. Although higher radon levels were measured in the homes of MS patients, the difference was not statistically significant, regional differences were demonstrated, and newly emerging patients are predominantly seen in high-risk regions [45].

It was reported that there was a correlation between high radon concentrations and MS [46]. Another study investigated the relationship between ionizing radiation sources and MS in two regions of Sweden. The findings illustrated a correlation between MS and X-ray exposure [47].

In Washington State, the highest prevalence rate of MS (225/100,000) was observed in Spokane County, which also had the highest radon exposure levels in the state. In contrast, King County, with the lowest prevalence rate (121/100,000), was identified as the region with the lowest radon exposure [48].

A study investigated the relationship between the indoor radon concentration and MS and reported that the incidence of MS was greater in areas with elevated radon gas levels. However, the difference was not statistically significant [7].

A study was conducted in the form of a small county-level analysis in northwestern Ireland and reported that counties with lower radon levels also presented lower estimated MS prevalence [49].

Three studies directly measured radon concentrations in the homes of participants with and without MS [50,51,52]. They measured radon gas concentration in homes with radon test kits and reported that individuals with MS were significantly more likely to live in homes with radon concentrations exceeding the World Health Organization’s standards (91.8%) than individuals without MS (8.2%). Similarly, some studies reported that the mean total concentration of radon gas was greater in the homes of MS patients, although the difference was not statistically significant [7,52].

A study reported that the levels of radon-222 (^222^Rn) and its decay product bismuth-214 (^214^Bi) were greater in the bedrooms of MS patients than in those of the controls, suggesting a potential link between radon exposure and MS. The same study recorded that the high-energy alpha and beta radiation originating from radon progeny particles is embedded within brain proteins in Alzheimer’s disease and brain lipids in Parkinson’s disease. They reported a tenfold increase in radioactivity in diseased brains compared with control brains. They hypothesized that the high-energy alpha and beta radiation from radon progeny embedded in brain tissues might play a role in the formation of the myelin sheath lesions observed in MS [53].

An animal study recorded that radon-exposed mice indeed show DNA damage corresponding to a calculated dose of 2.3 mGy, which corresponds to an effective low dose of ionizing radiation that might be capable of inducing changes in the brain’s dynamic [54].

Lykken and Momčilović proposed that myelin sheath lipids take up inhaled lipid-soluble environmental radon, subsequent α- and β-particle bombardment irreversibly damages myelin cell nuclei, puncturing the myelin sheaths beyond the point of repair and causing permanent nerve impulse propagation failure [55]. Radiation produces free radicals, which cause damage by peroxidation in the lipid portion of the myelin sheath [56]. 

Radon gas is not only an environmental risk factor for MS and other neurodegenerative diseases but also may be associated with lung cancer; thyroid, skin, and kidney cancer; and lymphoblastic leukemia in children [57,58,59,60].

### 4.1. Possible Mechanism for Radon

Radon, a byproduct of the radioactive decay of uranium, is easily released from soil and rocks, where it can fill voids along fissures, fractures, and faults. Since igneous rocks typically contain more uranium than metamorphic or sedimentary rocks do, areas with widespread igneous rocks often exhibit higher radon concentrations. The results of the research conducted by (Unpublish data) in the study area showed that igneous rocks such as tuff and trachyte have high uranium, thorium, and potassium values (Table 4) in higher radon areas such as İhsaniye city center, Kayıhan, Kemerkaya, and Döğer. However, sedimentary and metamorphic rocks such as alluvium, limestone, schist, and marble are characterized by low uranium, thorium, and potassium values in lower radon areas such as Gazlıgöl, Yaylabağı, Dişli, and Özburun.

The movement and velocity of radon in soil are influenced by the amount of water present in the soil pores. Radon migrates more efficiently in permeable, coarse-grained environments such as gravel than in impermeable soils such as clay. Fractures in soil and rock further facilitate the rapid movement of radon. In the air, radon moves faster than it does in water. Geological features significantly affect both the concentration and mobility of radon, resulting in variations in radon levels in dwellings depending on their location [6,61,62]. 

Radon primarily enters the human body through the inhalation of radon gas and the ingestion of water contaminated with radon [63,64]. After exposure, radon can enter the circulatory system, where it is distributed throughout the body [65]. This enables radon to cause both organ-specific and systemic health effects. Its radioactive properties result in cellular injury and DNA damage, significantly increasing the risk of developing cancer [66]. Thus, radon is a well-established carcinogen and represents the leading environmental cause of cancer-related mortality in North America [66]. Radon is most widely recognized for its role in increasing the incidence of lung cancer and has been identified as the primary cause in 3–20% of cases [67,68]. 

It was proposed that myelin sheath lipids absorb inhaled lipid-soluble environmental radon. This results in alpha and beta particle bombardment, causing irreversible damage to the nuclei of myelin cells and leading to permanent nerve impulse propagation failure. Additionally, the radiation-induced free radicals generated in this process cause peroxidative damage to the lipids in myelin, further compromising the structural integrity of the myelin sheath [69].

### 4.2. Limitations and Advantages of This Study

One of the main limitations of this study is the small population living in areas with radon concentrations below 300 Bq. Another limitation is that many environmental factors that may be effective in MS pathogenesis may be confounding factors. These unexamined factors could act as confounders and influence the results.

This study is important because it is the first to investigate the effect of radon gas on the prevalence of MS in Turkey. The majority of studies on radon gas have been registry-based or have compared radon gas in the homes of patients and controls. However, in this study, all the scanned areas were compared according to their seismic activity, divided into two regions, and measured with randomly distributed detectors. In this respect, the methodology of this study is more reliable.

## 5. Conclusions

The results of this study indicated that radon gas is an environmental factor in the etiopathogenesis of multiple sclerosis. Furthermore, high radon gas concentrations in areas with active fault lines and volcanic rocks indicate that living in these areas is an environmental risk factor.

Additionally, living in areas with active seismic activity could be considered an indirect risk factor for MS. However, the number of studies conducted on this topic is very limited, and the findings are often contradictory. Further research is needed to shed light on this potential relationship.

## Figures and Tables

**Table 1:** The source and distribution of radon in the study area.

Region	Radon Sources		Radon Distribution (Bq/m^3^)			**MS Prevalence** **per 100,000**
	Geology	Type of Fault	Minimum	Maximum	Average ± S.D.	
City Center of Bolvadin	Alluvium Bolvadin fault	Active fault	21	1325	307 ± 5.48	70.3
Kemerkaya	Seydiler ignimbirite Adatepe andesite	-	66	1603	350 ± 4.9	0
Kayıhan	Seydiler ignimbirite	-	65	1688	407 ± 4.45	0
City Center of İhsaniye	Seydiler ignimbirite	-	45	1878	399 ± 5	29.7
Döğer	Seydiler ignimbirite	-	69	941	314 ± 7.44	0
Dişli	Alluvium Dişli fault	Nonactive fault	27	489	192 ± 4.5	0
Yaylabağı	Köprülü volcano-sedimentary unit Gazlıgöl fault	Nonactive fault	61	447	182 ± 4.21	0
Gazlıgöl	Afyon metamorphics Gazlıgöl fault	Nonactive fault	12	607	162 ± 3.73	56.3
Özburun	Köprülü volcano-sedimentary unit Dişli fault	Nonactive fault	68	220	159 ± 11.49	34.1

**Table 2:** Demographical and clinical findings of the patients.

Baseline Characteristics	Mean Values
Age (years)	42.5 ± 15.7
Female/male (n)	10/4
RRMS/SPMS/PPMS (n)	10/3/1
Disease duration (years)	10.0 ± 10.9
Disease onset (years)	30.7 ± 8.3
EDSS (mean)	3.4 ± 1.8

**Table 3:** Initial symptoms of the clinically definite MS patients; number of patients.

Symptoms	Number of Patients
Pyramidal	4
Cerebellar	3
Brainstem	2
Sensory	5
Visual	2
Autonomic	1
Mental	4

**Table 4:** The average data (Bq/kg) of uranium, thorium, and potassium of rock samples from the study area.

Rock Types	Uranium	Thorium	Potassium
Alluvium	62.49	5.33	7.83
Limestone	3.54	4.98	5.85
Trahcyte	351.77	44.52	304
Tuff	264.51	53.63	1275.97
Schist	6.16	2.2	52.37
Marble	143.51	14.94	0

## Data Availability

The data supporting the findings of this study are available upon request from the corresponding author, F.İ.
